# The meaning of a very positive birth experience: focus groups discussions with women

**DOI:** 10.1186/s12884-015-0683-0

**Published:** 2015-10-09

**Authors:** Annika Karlström, Astrid Nystedt, Ingegerd Hildingsson

**Affiliations:** Department of Nursing, Mid Sweden University, Holmgatan 10, SE-70, Sundsvall, Sweden; Department of Nursing, Umeå University, Umeå, Sweden; Department of Women’s and Children’s Health, Uppsala University, Uppsala, Sweden

**Keywords:** Focus groups, Positive birth experience, Thematic analysis

## Abstract

**Background:**

The experience of giving birth has long-term implications for a woman’s health and wellbeing. The birth experience and satisfaction with birth have been associated with several factors and emotional dimensions of care and been shown to influence women’s overall assessment. Individualized emotional support has been shown to empower women and increase the possibility of a positive birth experience. How women assess their experience and the factors that contribute to a positive birth experience are of importance for midwives and other caregivers. The aim of this study was to describe women’s experience of a very positive birth experience.

**Method:**

The study followed a qualitative descriptive design. Twenty-six women participated in focus group discussions 6–7 years after a birth they had assessed as very positive. At the time of the birth, they had all taken part in a large prospective longitudinal cohort study performed in northern Sweden. In the present study, thematic analysis was used to review the transcribed data.

**Results:**

All women looked back very positively on their birth experience. Two themes and six sub-themes were identified that described the meaning of a very positive birth experience. Women related their experience to internal (e.g., their own ability and strength) and external (e.g., a trustful and respectful relationship with the midwife) factors. A woman’s sense of trust and support from the father of the child was also important. The feeling of safety promoted by a supportive environment was essential for gaining control during birth and for focusing on techniques that enabled the women to manage labour.

**Conclusion:**

It is an essential part of midwifery care to build relationships with women where mutual trust in one another’s competence is paramount. The midwife is the active guide through pregnancy and birth and should express a strong belief in a woman’s ability to give birth. Midwives are required to inform, encourage and to provide the tools to enable birth, making it important for midwives to invite the partner to be part of a team, in which everyone works together for the benefit of the woman and child.

## Background

The experience of giving birth has long-term implications for a woman’s health and wellbeing [1, 2]. Satisfaction with birth has been associated with several factors and the emotional dimensions of care have been shown to influence women’s overall assessment. Individualized emotional support empowers women and increases the possibility of a positive birth experience [3]. Support from the midwife during labour and birth and the opportunity to participate in decision-making are also important [4].

Woman’s perceived control during birth is significant [5, 6]. Findings from a mid-European study show that the most important factor contributing to a woman’s satisfaction with birth was having her expectations fulfilled. Self-efficacy and personal control were other factors related to satisfaction with birth. The experience of personal control had a protective effect on labour pain [7]. Dutch women assessed their birth experience as very happy when answering a questionnaire where five options were available; from very happy to very unhappy. In a description of caregivers, the most frequently chosen positive adjectives were supportive and considerate [8]. In a prospective, longitudinal study, 303 women (32.7 %) had a very positive birth experience assessed on a five-point Likert scale ranging from very positive to very negative [9].

Furthermore, women’s views of and satisfaction with maternity care are important indicators of quality service provision [10]. A recent major focus has been to identify the prevalence of (and associated factors for) a negative birth experience in order to improve care. Although equally as important, research relating to the factors associated with a positive birth experience have been less well studied. Learning from women’s birthing stories and adapting care during birth based on what women actually consider important will likely enhance the birth experience and promote normality in the context of the much medicalized birth environment.

Obstetric outcome is a major measure of the quality of care provided. However, how women assess their experience and the factors that contribute to a positive birth experience are also of importance for caregivers. The aim of this study was to describe women’s experience of a very positive birth experience.

### Obstetric context

Antenatal care in Sweden is organized within the public primary health care system with the midwife as the primary caregiver, providing care for pregnant women within a certain geographical area. Care during labour, birth and the postnatal period occurs in hospitals with midwives as the independent caregiver for uncomplicated cases. A nurse assistant is intermittently present during labour and birth and assists the midwife with practical care and support of the birthing woman. Midwives work in collaboration with obstetricians if complications occur. There are few alternative birth settings in Sweden and continuity of caregiver between episodes of care is rare. The prevalence of home births for Sweden is about one per thousand.

## Methods

### Design

This project was part of a large prospective longitudinal cohort study undertaken in northern Sweden in 2007. Data was initially collected by self-administered questionnaires. Women were approached to participate in the study after their ultrasound examination at gestational week 17–19 and subsequently followed-up at late pregnancy (32–34 weeks) and at 2 and 12 months post-partum. The questionnaires covered a wide range of questions, including questions addressing socio-demographic and obstetric background, attitudes, expectations and experiences related to childbirth. Answers to two questions were the inclusion criteria for participation in the study reported here. The first question, concerning women’s perception of their birth experience, was collected at 2 and 12 months post-partum. The second question was included in the third questionnaire where women agreed to be contacted for an interview. Women’s perception of birth was assessed on a five-point Likert scale ranging from very positive to very negative. A detailed description of the methods in the longitudinal cohort study is presented elsewhere [11].

### Participants

Women who had assessed their birth as very positive at 2 months post-partum and consented to participate in an interview were invited to take part in focus group discussions. A letter of invitation was sent to 197 women.

### Focus groups interviews

Interviews were performed between September 2013 and February 2014. The timing of the interviews was chosen partly due to practical reasons but foremost in the belief that women need time to process their experience. Focus group discussions were held in a conference setting at the university or at the primary health care center nearby to where women lived. The discussions lasted 2–3 h and were audio taped. Two researchers were present at each interview; one of the researchers started the session with a short presentation of the project and the other took notes. The participants were asked to present themselves and talk shortly about their present situation. The discussion started with an open-ended question: “Please tell us about your very positive birth experience and the importance of it.” Women were specifically asked to reflect on a birth that occurred in 2007 or 2008 (index birth).

### Thematic analyses

A qualitative descriptive approach was used in this study [12]. Thematic analyses were used to analyze the transcribed focus group data [13]. Three researchers thoroughly reviewed the transcripts to gain a fuller sense of their meaning. Initial concepts that emerged were discussed and coding was performed to identify patterns of words or statement that were related to the aim of the study. Next, all codes were examined and compared to clarify relationships, and the various codes were sorted into sub-themes. The intention during this process was to stay close to the words through a constant movement between the whole and the parts of the text. The accuracy of how to sort codes with similar content into sub-themes was confirmed in discussions in which all co-authors participated and agreed. In the final step, two themes were formulated to describe women’s very positive birth experiences.

All researchers are midwives with long experience of intra-partum care. The researchers paid careful attention throughout the interviews and analyses to the pre-understanding that inevitably will influence the interpretation of women’s birth stories.

### Ethical considerations

The prospective longitudinal cohort study was approved by the Ethical Committee at Umeå University (05–134 Ö). The approval for the larger prospective longitudinal cohort study included this study. In the third questionnaire women gave a written consent to be contacted for an additional interview. The invitation letter sent to the women made it clear that the time elapsed since their approval of participation in interviews was of importance. For example, things might have happened that made it no longer possible for the women to participate. The women were also assured that, at any time, they could withdraw from the focus group’s discussion. Several women had given birth after the index birth but none of them were pregnant at the actual focus group session.

## Results

Initially, 197 of the 212 eligible women fulfilled the inclusion criteria and were sent a letter of invitation to participate in focus group discussions. Of the 59 responses, 30 women agreed to take part, with 26 of these fulfilling the appointment. Seven focus groups were carried out with 2–6 participants in each group. Women were aged between 28 and 46 years with a mean age of 36. They were all born in Sweden, married or cohabiting and gave birth in hospital. Most women had a higher level of education (18/26). The majority of women gave birth vaginally except two women who had an emergency caesarean section. There were 9 primiparous women in the study group.

Six to 7 years after delivery, all women looked back very positively on their birth experience. Women spoke mostly about the birth they had assessed as very positive, but comparisons were also made about previous or later births. Two themes and six sub-themes were identified that described the meaning of a very positive birth experience, as shown in Table 1.

**Table 1:** Themes and sub-themes

Themes	Sub-themes
Experiencing own ability and strength	Feelings of confidence with positive expectations
	Physical and mental preparation
	Control gives ability to relax and let go
Having trustful and supportive relationships	Feelings of safety, be seen and heard
	Midwife as a guide
	Teamwork with parents and staff

Many expressions were used to describe the birth experience. Frequently used words included fantastic, wonderful, magic, incredible and overwhelming. When women were asked to further develop the feelings they had experienced during birth, they spoke about being in a flow, fully engaged and participating, feeling safe and secure, close to the earth, experiencing happiness through a very special journey, and an important moment of life. The women spoke with pride at having coped with the labour and pain, which made them feel thankful and relieved. They also talked about child birth as an outstanding experience that they felt positive about experiencing again.

### Experiencing own ability and strength

The first theme is an expression of a general attitude among the women that became evident when listening to the birth stories. The women's approach towards childbearing was grounded in an attitude of trust in themselves and their ability to give birth. Feelings of confidence were clearly expressed in all focus groups and the women were convinced that they were able to handle the pain and other difficulties. It was suggested that their positive birth experience was related to their constructive and sympathetic attitude towards childbearing and life in general.

Mother and grandma have given birth; it is in our nature to do this. So it felt like I had a basic attitude that it would be simple. And it was. (First child, vaginal birth)

Or is it not so you are a certain person to have a positive birth? It doesn’t start with giving birth, but it is a general attitude. (Third child, vaginal birth)

Most of the participants believed this confidence led to positive expectations towards the coming birth. There was an agreement among participants in the focus groups that giving birth was something they looked forward to, and they were inspired by mothers, grandmothers and other important females held up as role models. To be pregnant and give birth were matters of course and a privilege. Birthing was described as a natural process for which the female body was designed, however, all of the focus groups agreed that expectations should be realistic. Knowledge about the birthing process and obstetric complications, such as prolonged labour and emergency caesarean, was considered necessary.

I had a model in my mother who had told me about her fine and normal births. I did not expect anything else. (Second child, vaginal birth)

It is a magic experience, but somehow you have to be aware of what it is about. When you talk about giving birth, I think it is important not to make it strange but to make it very concrete. (Second child, vaginal birth)

Physical and mental preparation was a necessity for most of the women, as was participation in classes. Individual preparation was also described as important. Women wanted to be taught about the practice of labour, including breathing and relaxation techniques, as well as how to be physically and psychologically prepared to give birth. They wanted their partners to be involved and acknowledged by the professionals. Physical and mental preparation, including knowledge about the birthing process, was necessary to achieve control and take part in decision-making. The women did not consider giving total responsibility to the midwife.

For me, the mental training was important…it was very good. We talked a lot about expectations, which is useful not only when giving birth, and about our relation that we do this together. (First child, emergency caesarean section)

I was prepared, I knew how to breath (during birth) and how to breastfeed. (Second child, vaginal birth)

Preparations before birth also meant that women were aware of potential obstetric complications and deviations from the normal birth process. A few women described a more expectant attitude towards birth and there was a general acceptance that pain and other difficulties were part of giving birth. Several women had experienced prolonged labour, emergency caesarean section or postpartum hemorrhage during the index birth. The pain was generally described as intense and stronger than expected, and was handled in different ways. Controlled breathing, mental training, and nitrous oxide were the most commonly used pain relief methods, with a few of the women having an epidural. The breast-feeding period was initially painful and troublesome for a few women, but irrespective of the origin of the pain, it was an expected challenge to overcome.

Something that is good to know before (you give birth) is that the pain is awfully intensive but then there is a break, between contractions you can really rest. (Second child, vaginal birth)

But you sort of forget and it is not a negative pain like an aching leg and not knowing for how long it will go on. It is a positive pain. (First child, vaginal birth)

The experience of control was mostly related to the management of pain but was also associated with the women having an active role during the course of birth. The women expressed feelings of being in charge and being involved in decision making. As important as control was the ability to let go, and the women discussed the need to relinquish control and to trust their body; to be in a relaxed mode and to await and work with the contractions. Control was related to the preparations that had been made prior to the birth. To be prepared and aware of the mental and bodily challenges involved in a birth made women more confident, as they knew what to expect and were more familiar with the birthing process.

I am sort of a control freak…my plan was to work with the breathing and mental training and it worked all the way. (First child, vaginal birth)

The thing I found so crucial was that you were in tune with your body, could listen to your body. That is what I mean with let go and find rest. (First child, vaginal birth)

### Having trustful and supportive relationships

The second theme expresses the importance of having caring and loving persons around birthing women. The presence of the child’s father was essential for most women in the focus groups, as this made the woman feel safer; by confirming that he believed in her and thus strengthening her self-confidence. The presence of a midwife or the assistant nurse was another significant person who made the birth positive.

Feelings of safety were repeated like a mantra throughout the discussions. The experience of safety was initially promoted by the very first telephone contact, when the women announced their arrival to the labour ward. It was important for the women to be met with interest from the midwife and not to be questioned about the severity of the labour. Several women expressed that it was a positive experience not to be turned away during the early phase of the labour. Feelings of safety were further confirmed by a respectful welcome and positive atmosphere in the birthing suite. Many of the women felt that giving birth was basically their own task and challenge, but the knowledge that they were in a place where they felt safe deepened their feelings of a very positive experience.

I was taken care of in a good way and felt safe, and yes, I think this is basic. (First child, vaginal birth)

When I think about this first birth and why it was so positive, it had much to do with the people around me in control of the complication I had (postpartum hemorrhage). Still I felt safe and calm. (First child, vaginal birth)

A trusting relationship involved feelings of being seen and heard, which meant that women experienced support on their own terms. The engagement from the midwife and the other staff enabled the women to feel safe and to be able to do what they wanted. They felt comfortable to move about, take a bath or simply moan through a contraction. This could also include an early admittance to the labour ward or not being questioned about positions during labour but instead being free to act in the way that felt best. Being seen and heard made these women confident that they had the leading role and that all considerations were made to prioritize them and their babies.

For me, a big part of the positive experience was that I felt seen and provided for, and that I was taken care of. (Second child, emergency caesarean section)

Yes, my birth was a quick one, but I think that the care and the reception you get are important. And that it clicks with those you are going to work with. (First child, vaginal birth)

In all of the focus groups, it became clear among most women that giving birth requires teamwork, where the woman and her partner work together with the midwife. This teamwork also included the assistant nurse and made it possible that care throughout labour and birth was directly related to the needs of the woman and her partner. Some women actively wanted and appreciated the staff being present to support and assist, whereas others only wanted the staff to be around if needed.

I mean, we were two sharing the pregnancy and it was a very positive time expecting the first child, so fun and exciting like the best journey you have been on. I felt safe with him (during birth) and there was nothing strange. (First child, vaginal birth)

I soon felt a sense of cooperation with the midwife and the assistant nurse. This was something we would do together. (Third child, vaginal birth)

The role of the midwife was discussed in all focus groups and women agreed on the importance of the midwife as a guide through labour and birth, and stressed the importance of being in tune with the midwife. The presence of the midwife in the room was reassuring for most women, although there wasn’t always a need for her to be active or instructive, but simply to be there. Confirmation and support from the midwife strengthened the woman’s own ability to stay in control and, at the same time, enabled her to relax. Other women were unconcerned about the midwife and were convinced that they would have managed on their own. The guidance of the midwife was essential if complications or difficulties occurred during birth.

I do not remember what they looked like or their names. At my first birth there was this old, very sweet midwife, but I could have been alone in the room. The staff was of no importance for me. (Third child, vaginal birth)

Afterwards when I thought about it, this midwife made the atmosphere in the room so very nice and cozy even when they had to remove the placenta and I had anesthesia and all that, I felt very positive about the birth. (Second child, vaginal birth)

The meaning of a very positive birth experience varied between women, where some women felt their own ability was the main factor and others describing the importance of support and help from the partner, the midwife and/or other staff involved. The citation below illustrates a general attitude in the focus groups. Women’s stories and the discussions about the meaning of a very positive birth experience focused on the birthing process. Still, it was understood and discussed as a matter of course that the birth of a child and becoming a mother was the foremost and central experience.

Yes, I just want to agree with you, the meaning of it all was the baby. And the treatment was good, but the best I think was that I was part of it, I was prepared and had made up my mind that it would be fine this time. (Second child, vaginal birth)

## Discussion

This study showed that women related their very positive birth experience to internal factors, such as their own ability and strength, as well as external factors, such as a trustful and respectful relationship with the midwife. A woman’s sense of trust and support from the father of the child was also important. The feeling of safety promoted by a supportive environment was essential to gaining control during birth and focusing on techniques enabling women to manage labour.

A variety of expressions were used in women’s birth stories to describe their very positive experiences. These expressions reflected a significant moment in the women’s lives, where feelings of joy and happiness dominated. Crowther et al. [14] studied the experience of joy at birth from a hermeneutic perspective and concluded that, although the literature on women’s birth experiences is extensive, it rarely focuses on joy. There is therefore a need to explore experiential aspects of birth beyond the type, place and outcome of the birth. The present study reports profound and reflective experiences narrated by the women and further developed along the discussions in the focus groups.

The women’s experience of their ability and strength was one of two overarching themes in this study. Birth specific domains most relevant for women’s overall experience have been identified that relate to this result. Seven domains were described in a mixed-method design study that included literature review, focus groups and consensus discussions with women and professionals [15]. The authors propose that the availability and quality of received care, concerns about safety, the first contact with the newborn, and health of the baby are key aspects for a mother’s overall birth experience. Further, the domain related to the health professional’s behavior and attitudes, as well as women’s feelings of confidence and safety, are of more importance than the course of labour, or the medical and environmental aspects of the birth. These findings are in line with the results of our study, where women were convinced that they would manage the challenges of giving birth. Maternal confidence e.g., a woman’s confidence in her ability to cope has been acknowledged in research. Self-efficacy can be defined as the confidence a person feels about performing a particular task [16]. Related to childbirth, Lowe [17] developed the Childbirth Women Self-efficacy Inventory, which has been used in several studies [18, 19]. Woman with increased self-efficacy experience less fear and pain and more satisfaction with birth compared to women with low self-efficacy.

Feelings of confidence and its relevance for positive expectations were clearly important among women in the focus groups. The fulfillment of expectations has been shown to be the most consistent determining factor of satisfaction with childbirth. The self-esteem of the woman, sense of accomplishment, and confidence as a new mother is further enhanced by positive perceptions and satisfaction with the birth experience [7, 20].

It is well known that women’s sense of control during childbirth is related to a positive birth experience [21, 22]. In a concept analysis, Meyer [23] identified four attributes of control in childbirth: decision-making, access to information, personal security, and physical functioning. This is consistent with the results of this study, and these aspects of a positive birth experience were all discussed in the focus groups. Women mostly described control in relation to bodily function and pain. Their ability to handle pain and other difficulties was a source of satisfaction that contributed to their positive experience. Women expected to be informed about the progression and other matters of importance; taking part in decision-making was discussed as natural unless it was an emergency situation. The meaning of control was mostly identified as an internal capacity, that is, the ability to maintain control over the behavior and the body. Other women talked about their experience of having influenced the atmosphere in the room and the behavior of the midwife, which can be considered external control [24]. The experience of control made it easier for the women in labour to turn their focus inward, letting go of the outside world. This was difficult unless women felt that they had taken command of the birth and this can be interpreted as maintaining control so as to avoid ‘losing oneself’ [25, 26].

Trustful and supportive relationships were essential for a positive birth experience. It was clear that the presence of the midwife was important even if the woman felt capable and did not explicitly need help and support from the midwife. The midwife and the assistant nurse ensured feelings of safety and calm for the birth mother, and the significance of these relationships with caregivers is evident in research [27, 28]. A systematic review [28] identified four key factors essential for women’s satisfaction with birth, with two of these being directly related to the mother-caregiver relationship: the quality of the relationship between the birthing mother and the caregiver; and the amount of support the woman received. The attitude and behavior of the caregivers expressed in the relationship with the woman outweighed the effects of all other variables (e.g., age, birth environment and medical interventions). Hunters’ study [29] on women’s experiences of birthing with doulas is of interest. Interviews with nine doulas and nine mothers revealed that the doulas, as paraprofessionals, were “holding the space”. This birthing space for women was created by components mentioned as crucial in the focus group’s discussions; the practical techniques and methods, the relationship, and the physical and emotional support. The mother-caregiver relationship with the midwife helped some women achieve an intimate space within an institutionalized birth and this was typically very much appreciated. Almost all births in Sweden take place in a hospital, which has become the natural and culturally accepted place to give birth, with only a few available alternatives (e.g., midwifery-led birth centers or home birth). It is crucial that a beneficial and supportive environment can be established within the walls of a medicalized maternity care.

The relationship with the father of the child was identified as another partnership of great importance for the women. Most women agreed that it was essential to have their partner close. The father effectively acting as a guard in the birthing room; well aware of the wishes and needs of the woman. Previous research demonstrates the importance of partner’s support on women’s birth experience. Among 325 Finnish first-time mothers, the attitude of the child’s father toward pregnancy was one of three predictors for a positive birth experience [30]. The partner’s support during birth was described as critical in helping women cope with labour by providing encouragement and reassurance [31]. Women in the focus groups described being part of a team, where all participants were significant for the birth experience.

This study is a further example amongst the literature showing that it is difficult to separate the birth experience from the care given [32]. Focusing solely on the experience will overlook the birthing environment and overlook the synergistic effect of a caring and reassuring midwife working together with the woman and her partner. In a recent study, women who stated that they were looked after very well and had a very positive birth experience were significantly more likely to have experienced high postnatal functioning [33]. This again highlights how it is possible to enhance a positive birth experience by developing maternity care customized to deliver what women want.

A principal strength of this study is its longitudinal design, where women first assessed their birth as very positive and then participated in focus group discussion six to seven years later, entering more deeply into the experience. To our knowledge, this has never been previously reported. All women had accurate and vivid memories of their birth. It is well known that women clearly remember their birth experience [34]. The decision to use focus groups for data collection proved accurate and effective, with all women taking an active part in the discussions.

However, the findings are limited to the data collected during seven focus groups in a Swedish setting. The participating women are a relatively homogenous group, which might have influenced the discussions. The timing of recruitment, 6–7 years after the index birth assessed as a very positive birth experience, is also of importance. Women who responded to the invitation had an interest in birthing experiences and a willingness to share them with others, despite the years that had passed, which might differentiate them from other women. It is difficult to generalize the findings, but we suggest that the result of the study may be transferable to other birthing women in a similar context. The interaction with and support from the birth partner and other caregivers is most likely a universal experience. The judgment of trustworthiness in the study should be based on transferability, credibility and dependability [35]. The presentation of data, the process of analysis, and the quotations from the transcripts confirm the credibility of the study. Dependability was established through ongoing discussions between the authors where agreements were made on the interpretation of data.

## Conclusion

It is an essential part of midwifery care to build relationships with women where mutual trust in one another’s competence is critical. The midwife is the active guide through pregnancy and birth and expresses a strong belief in a woman’s ability to give birth. Midwives are required to inform and encourage women, and to provide tools to overcome the challenge of birth. It is important for midwives to invite the partner to be part of a team in which everyone works for the best interest of the woman and child.

## References

[1] Simkin P (1991). Just another day in a woman’s life? Part I Women’s long-term perception of their first birth experience. Birth.

[2] Crompton J (2003). Post-traumatic stress disorder and childbirth.

[3] Nilsson L, Thorsell T, Hertfelt Wahn E, Ekström A (2013). Factors influencing positive birth experiences of first-time mothers. Nurs Res Pract.

[4] Hodnett ED, Gates S, Hofmeyr GJ, Sakala C (2011). Continuous support during childbirth. Cochrane Database Syst Rev.

[5] Dencker A, Taft C, Bergqvist L, Berg M (2010). Childbirth experience questionnaire (CEQ): development and evaluation of a multidimensional instrument. BMC Pregnancy Childbirth.

[6] Melender HL (2006). What constitutes a good birth? A qualitative study of pregnant Finnish women. J Midw Women’s Health.

[7] Christiaens W, Bracke P (2007). Assessment of social psychological determinants of satisfaction with childbirth in a cross-national perspective. BMC Pregn Childbirth.

[8] Rijnders M, Baston H, Shönbeck Y, van der Pal K, Prins M, Green J (2008). Perinatal factors related to negative or positive recall of birth experience 3 years postpartum i Netherlands. Birth.

[9] Hildingsson I, Johansson M, Karlström A, Fenwick J (2013). Factors associated with a positive birth experience: an exploration of swedish women’s experiences. Int J Childbirth.

[10] Jackson JL, Chamberlin J, Kroenke K (2001). Predictors of patient satisfaction. Social Sci Med.

[11] Hildingsson I, Thomas J, Karlström A, Olofsson Engström R, Nystedt A (2010). Childbirth thoughts in mid-pregnancy: prevalence and associated factors in prospective parents. Sex Reprod Healthc.

[12] Sandelowski M (2000). Whatever happened to qualitative description?. Res Nurs Health.

[13] Burnard P, Gill P, Stewart K, Tresure E, Chadwick B (2008). Analysing and presenting qualitative data. Br Dent J.

[14] Crowther S, Smythe E, Spence D (2014). The joy at birth: An interpretive hermeneutic literature review. Midwifery.

[15] Gärtner F, Freeman L, Rijnders M, Middeldop J, Bloemenkamp K, Stiggelbout A (2014). A comprehensive representation of the birth-experience: identification and prioritization of birth-specific domains based on a mixed-method design. BMC Pregnancy Childbirth.

[16] Self-efficacy BA (1977). The exercise of control.

[17] Lowe NK (1991). Maternal confidence in coping with labor: a self-efficacy concept. J Obstst gynecol neonatal Nurs.

[18] Lowe NK (2000). Self-efficacy for labor and childbirth fears in nulliparous pregnant women. J Psychosom Obstet Gynaecol.

[19] Beebe KR, Lee V, Carrieri-Kohlman HJ (2007). The effects of childbirth self-efficacy and anxiety during pregnancy on prehospitalization labor. J Obstet Gyn Neonatal Nurs.

[20] Kringeland T, Daltveit AK, Møller A (2010). What characterizes women who want to give birth as naturally as possible without painkillers or intervention?. Sex Reprod Healthc.

[21] Green J, Baston H (2003). Feeling in control during labor: concepts, correlates, and consequences. Birth.

[22] Goodman P, Mackey MC, Tavakoli AS (2004). Factors related to childbirth satisfaction. J Adv Nurs.

[23] Meyer S (2012). Control in childbirth: a concept analysis and synthesis. J Adv Nurs.

[24] Bryanton J, Gagnon AJ, Johnston C, Hatem M (2008). Predictors of women’s perceptions of the childbirth experience. JOGNN.

[25] Parrat J, Fahy K (2003). Trusting enough to be out of control: a pilot study of women’s sense of self during childbirth. Aus J Midwifery.

[26] Lundgren I (2005). Swedish women’s experience of birth 2 years after birth. Midwifery.

[27] Lundgren I, Berg M (2007). Central concepts in the mid-wife relationship. Scand J Caring Sci.

[28] Hodnett E (2002). Pain and women’s satisfaction with the experience of childbirth: a systematic review. Am J Obstet Gynecol.

[29] Hunter C (2012). Intimate space within institutionalized birth: women’s experiences birthing with doulas. Anthropol Med.

[30] Tarkka MT, Paunonoen M, Laippala P (2000). Importance of the midwife in the first-time mother’s experience of childbirth. Scand J Caring Sci.

[31] Gibbins J, Thomson AM (2001). Women’s expectations and experiences of childbirth. Midwifery.

[32] Larkin P, Begley CM, Devane D (2009). Women’s experience of labour and birth: an evolutionary concept analysis. Midwifery.

[33] Michels A, Kruske S, Thompson R (2013). Women’s postnatal psychological functioning: the role of satisfaction with intrapartum care and birth experience. J Reprod Infant Psych.

[34] Takehara K, Noguchi M, Shimane T, Misago C (2014). A longitudinal study of women’s memories of their childbirth experiences at five years postpartum. BMC Pregnancy Childbirth.

[35] Lincoln YS, Guba EG (1985). Naturalistic inquiry.

